# Multiomic Characterization of Male and Female Aging Brain in Aromatase Deficiency Mice at Single‐Cell Resolution

**DOI:** 10.1002/alz.095491

**Published:** 2025-01-09

**Authors:** Tanvi Potluri, Guadalupe Rodriguez, Hongxin Dong, Hong Zhao, Serdar E Bulun

**Affiliations:** ^1^ Northwestern Feinberg School of Medicine, Chicago, IL USA; ^2^ Northwestern University Feinberg School of Medicine, Chicago, IL USA

## Abstract

**Background:**

Aging and the decline in sex steroid hormone (e.g., estrogen) are associated with a potential loss of its neuroprotective effects on the female brain. Aromatase, a pivotal enzyme in the synthesis of estradiol (E2), is highly expressed in various brain regions, notably the hippocampus. However, how estrogen regulates brain function and how dysregulation of estrogen signaling modulates the risk of Alzheimer’s disease are not well understood. Furthermore, the mechanisms through which E2 modulates brain cell‐type specific transcriptomic and epigenetic changes, particularly in the context of aging and susceptibility to Alzheimer’s disease, remain inadequately understood. Additionally, investigations into the effects of E2 on both male and female sexes are limited.

**Method:**

In this study, we employed a brain‐specific aromatase knockout (bArKO) mouse model to examine the epigenetic and transcriptomic alterations in mouse brains characterized by E2 deficiency and aging. We performed single‐nucleus RNA sequencing (snRNA‐seq) and Assay for Transposase‐Accessible Chromatin with sequencing (snATAC‐seq) at the same cells from the hippocampus of ∼20‐month‐old male and female bArKO mice and their wild‐type (WT) littermate controls (n = 3/sex/genotype).

**Result:**

Overall, 77,200 nuclei were analyzed post quality control, annotating 32,285 genes. After dimension reduction, we obtained 23 clusters representing excitatory and inhibitory neurons, microglia, astrocytes, oligodendrocytes, oligodendrocyte precursor cells, ependymal cells, and perivascular macrophages. Four populations of *Gad1* ^+^ inhibitory neurons were observed: *Sst* ^+^, *Vip1* ^+^, *Crispld1* ^+^, and *Cdh18* ^+^. Conversely, eight populations of excitatory neurons were uncovered, with significant transcriptomic changes observed between WT and bArKO neurons. Pathway analysis revealed overrepresentation of dopamine secretion regulation and dendrite arborization in bArKO mice. Additionally, known sex‐specific genes were observed. Males predominantly expressed *Eif2s3y* and Uty while female mice showed higher expression of *Tsix*, *Xist*, and *Jpx*.

**Conclusion:**

In summary, our study sheds light on the molecular and cellular alterations occurring in the hippocampus of aging mice with E2 deficiency, particularly focusing on the role of aromatase in modulating brain function. The identification of distinct neuronal populations and differential gene expression patterns between WT and bArKO mice highlights the complexity of E2‐mediated neuroprotection and its relevance in both sexes.

